# Fluorescence videography for the rapid and automated in-clinic detection of microfilariae

**DOI:** 10.1186/s13071-026-07486-y

**Published:** 2026-08-20

**Authors:** Britt Ripley, Mackenzie Smith, Alex Chen, Kevin Calcote, Andrew R. Moorhead, Elyssa Campbell, Paul Slusarewicz

**Affiliations:** 1 Parasight System Inc., Suite 2130, 1532 N. Limestone St., Lexington, KY 40505 USA; 2 University of Kentucky, M.H. Gluck Equine Research Center, Lexington, KY 40546 USA; 3 Department of Clinical Sciences, College of Veterinary Medicine, North Carolina State University, Raleigh, NC 27606 USA; 4 Department of Infectious Diseases, College of Veterinary Medicine, University of Georgia, Athens, GA 30602 USA

**Keywords:** *Dirofilaria immitis*, Heartworm, Microfilaria, Veterinary diagnostics, Parasight All-in-One

## Abstract

**Background:**

Heartworm infection is a serious cause of both morbidity and mortality in canids. Current guidelines from professional bodies recommend annual testing using both molecular (ELISA/PCR/Lateral Flow) and microscopic (detection of microfilariae in blood) testing. The inconvenience of the latter, however, presents a barrier to some clinicians towards adherence to these guidelines. We have recently developed a fully automated, rapid and hands-free test that uses videography coupled to computer vision analysis to detect and enumerate blood-borne microfilariae.

**Methods:**

Here we describe the performance of this test compared to a common clinical option – the 20 μL wet mount. Multiple counts were conducted by both methods on serially diluted infected blood to determine count correlations and on highly diluted and negative samples to compare sensitivities and specificities.

**Results:**

Automated counts correlated well (*R*^2^ = 0.9851) at microfilarial concentrations of up to 1000 microfilariae/mL, after which the automated counts began to plateau as microfilariae began to overlap and became more difficult to distinguish from each other. Within the linear range, the automated method counted more than 10-times as many microfilariae in the same blood samples than the manual method; this translated to a sensitivity of 100% at approximately 50 microfilariae/mL compared to 60% for the manual wet mount. After a further 2-fold dilution the sensitivity of the manual method dropped to 10% while that of the automated remained at 100%.

**Conclusion:**

These results indicate that the automated system is a viable option to replace current manual options in-clinic while combining superior sensitivity with greater convenience.

**Graphical Abstract:**

**Figure:**
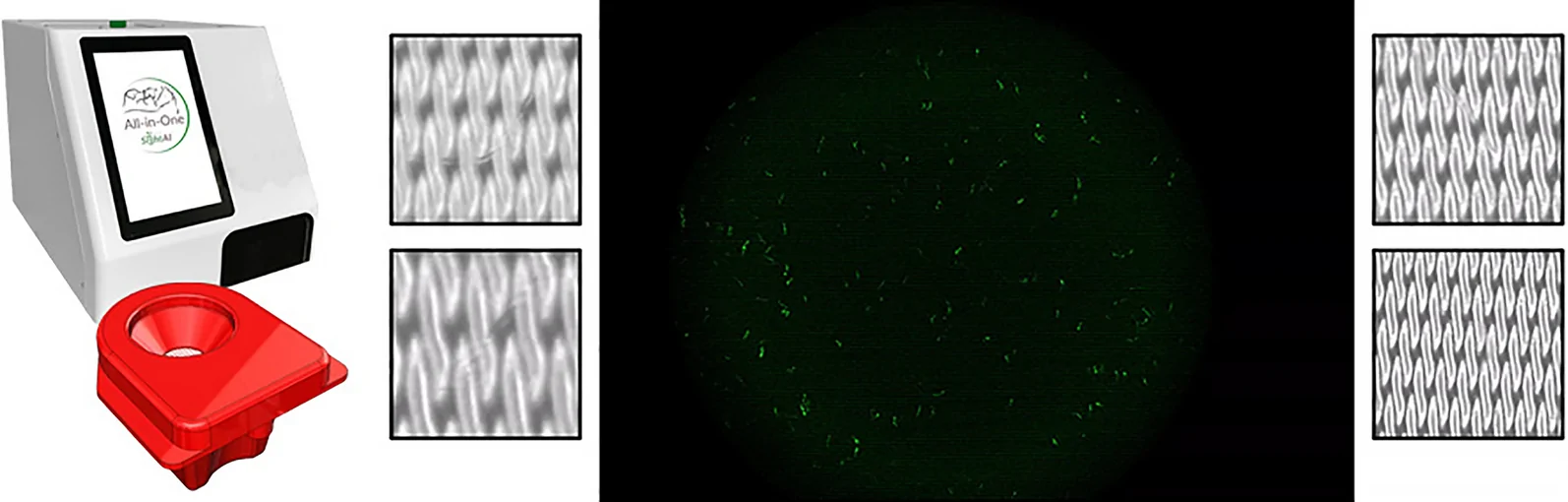

**Supplementary Information:**

The online version contains supplementary material available at 10.1186/s13071-026-07486-y.

## Background

Filarioids are a globally distributed group of nematode parasites [1] that infect both humans and other animals via intermediate hemophagous arthropod vectors [2]. Cases of filariasis are increasing globally owing to the spread of mosquito vectors into emerging ecological niches owing to increasing temperatures and humidity driven by anthropogenic climate change [3–5]. In the case of veterinary medicine, *Dirofilaria immitis* and *D. repens* are major filarid pathogens of canids and, to a lesser extent, felids [6]. Infections in canids are primarily diagnosed by examining blood using either immunological, molecular or microscopic methods [7–9].

Microscopic methods entail the visual detection of microfilariae in blood, which positively indicates patent infection. Microfilariae can be observed in a simple blood smear or wet mount, although such methods are somewhat insensitive owing to small sample sizes (~ 20 µL). Alternative methods have therefore been developed that use more blood (~ 60 µL); these include examination of hematocrit tubes post-centrifugation, where the microfilariae can be found in the buffy coat layer [14], and the use of specialized counting chambers [15], although these provide only modest improvements in sensitivity. Two tests have been developed to maximize the sensitivity of microfilarial detection by examining larger volumes of blood (typically 1 mL). Using larger blood samples increases the likelihood of detecting infections, especially in cases where microfilariae are present in low numbers.

Immunological methods include ELISA tests [10], which are too complex for in-clinic use and so are generally offered as services by reference laboratories, or lateral-flow tests that use a similar antibody sandwich concept but are much simpler and can be used in-clinic with minimal need for equipment or training [11]. Molecular methods consist of conventional and multiplexed polymerase chain reaction (PCR) assays [12, 13], which are exclusively used in reference laboratories.

In less sensitive tests such as wet mounts, the eye-catching motility of the microfilariae aids in their detection under the microscope; however, the hemolytic steps used in the more sensitive Knott and filter tests either kill or pacify the microfilariae before they can be examined, making them more difficult or observe. As a result, histological stains such as methylene blue or Giemsa are used to enhance the visibility of the microfilariae [16, 17]. Moreover, microfilariae have been stained with fluorescent probes, such as acridine orange [18] or a variety of fluoresceinated lectins [19–22], to render them more visible to the analyst. However, most veterinary practices lack a fluorescence microscope to perform such analyses, rendering them unsuitable for routine use in clinical practice.

In both of the more sensitive methods (Knott and filter), the blood is first hemolyzed either by osmotic shock and/or by using a surfactant. In one, the Knott test [23, 24], the hemolyzed sample is centrifuged to sediment and concentrate any microfilariae present. The resulting pellet is then examined microscopically. However, the pellet is usually 200 µL in volume and so requires multiple slides to be counted manually, which is a time-consuming process. In the other method, the hemolyzed sample is passed through a fine membrane filter (usually with a pore size of 5 µm) to concentrate and trap the microfilariae before evaluation [25]. The filter is then examined for the presence of microfilariae.

Both of these more sensitive methods are labor-intensive and slow, primarily because of the time required for examination of samples at moderate-to-high magnification and the additional 10 min needed for hemolysis. All these tests require well-trained analysts who are motivated to perform the monotonous task of scanning slides manually with high fidelity. These requirements are not well-suited for use in a clinical practice that sees multiple patients simultaneously and requires results while those patients are still in the clinic so that anthelmintic treatments can be prescribed or administered as soon as possible. Furthermore, in the case of veterinary medicine, professional bodies, such as the American Heartworm Society and the Companion Animal Parasite Council, recommend that both antigen and microscopic methods are employed in diagnosis [9]; however owing, in part, to the inconvenience of microscopic methods, including the less onerous less sensitive methods, some veterinarians rely on molecular methods alone, potentially risking missing infections [26].

The Parasight System is a veterinary parasitology diagnostic device originally developed to detect and enumerate the ova and oocysts of a variety of gastrointestinal parasites in fecal samples [27–31]. The system functions by labeling these parasitic products with fluorescent markers and digitally imaging samples using a fluorescence-based optical column prior to computation image analysis to identify and enumerate objects of clinical interest.

Here we describe a new test we have developed to utilize the capabilities of this device for the detection of *D. immitis* microfilariae in whole blood using fluorescence videography and compare its performance to a standard wet mount—the simplest method currently available to veterinarians in-clinic.

## Methods

### Samples

Uninfected dog blood was obtained from the Filariasis Research Reagent Resource Center at the University of Georgia. *Dirofilaria immitis* microfilaremic dog blood, generally containing 30,000–60,000 microfilariae per mL (mf/mL), was obtained from purpose-bred beagles at the University of Georgia. A single infected dog was used for positive samples (approximately 3–5 mL drawn at any given time) and a single uninfected animal was used for the negative sample (approximately 10–15 mL drawn at any given time).

Samples from the same infected and uninfected dogs were used throughout the study, although fresh samples were used in each experiment. Samples were shipped on ice on Mondays, received on Tuesdays, and each experiment (other than the longevity study) was conducted and completed by Friday. All blood was collected in purple-cap ethylenediaminetetraacetic acid (EDTA) tubes and samples were stored at 4 °C when not being used.

### Wet mount counting

Blood samples were gently inverted multiple times to disperse settled cells and microfilariae immediately prior to placing 20 µL onto a glass slide, which was then overlaid with a glass coverslip. The entire surface area of the cover slip (including the blood that had exuded at its edges) was examined manually at 100 × magnification; all the observed microfilariae were counted. Manual counts were performed by two analysts, who had previously been cross-trained to the point that their counts on the same training slides were consistently within 5% of each other.

### Automated counting

**Fig. 1:**
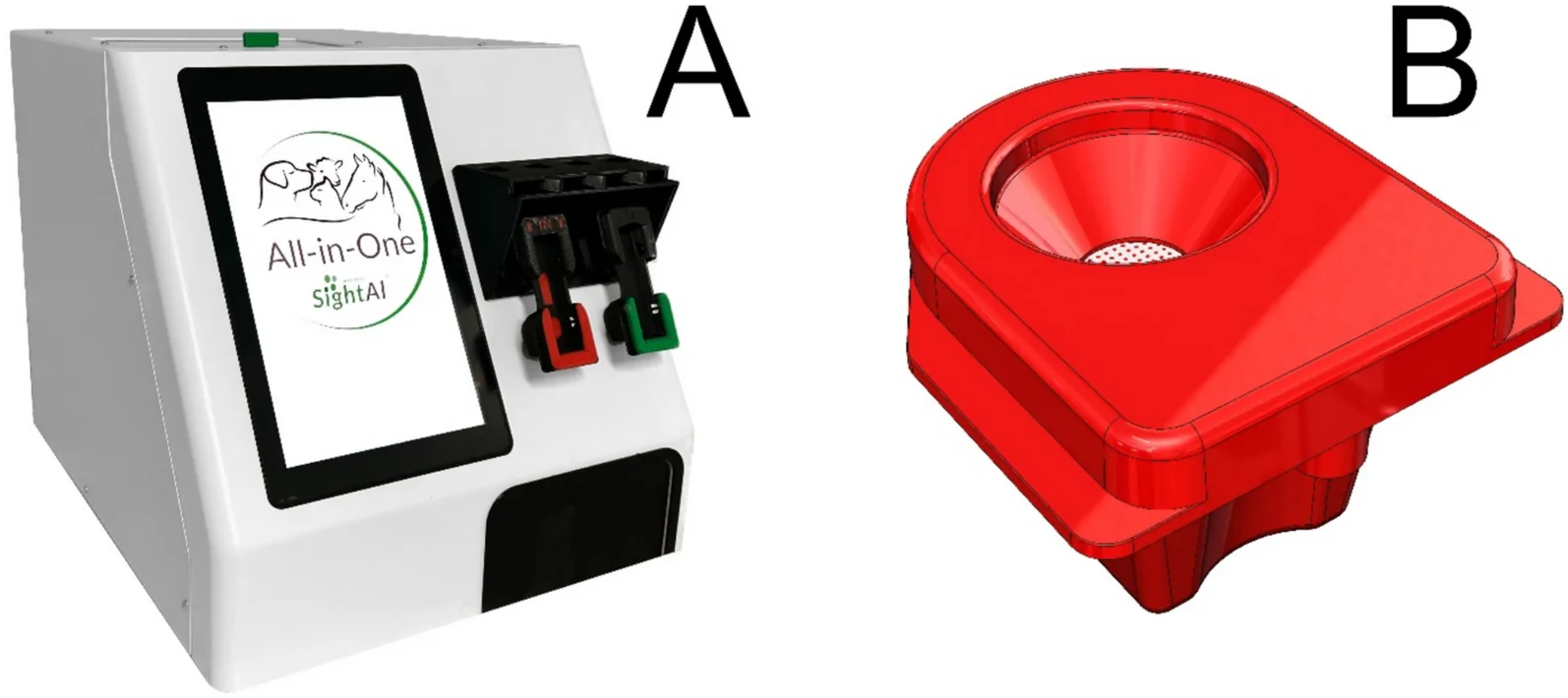
The Parasight All-in-One device (**A**) and single-use sample chamber (**B**)

Automated counting was conducted using the Parasight All-in-One (AIO) parasitology diagnostic device (Fig. 1A). Each test uses a single-use plastic sample chamber (SC; Fig. 1B). The SC is comprised of a lower and upper half that are welded together; the lower half contains a waste receptacle to capture the sample and various solutions required for testing, while the upper half contains an integrated stainless-steel mesh (Φ = 10 mm) to capture analyte particles and a vacuum port used to draw liquids through the mesh.

Samples were processed and imaged in the Parasight All-in-One unit. The unit’s imaging column consists of: an array of blue LEDs for fluorescence illumination; a custom-made macro lens (i.e., 1 × magnification); a green long-pass emission filter integrated into the lens; and a digital camera with an 18 MP monochrome sensor.

 One mL of blood was added to 5 mL of deionized water and mixed gently by inversion. The sample was then immediately poured onto a 5 µm Dutch weave stainless steel mesh integrated into the SC and drawn through with gentle vacuum. The sample was then washed three times with 0.2 mL of phosphate-buffered saline (PBS). Following the addition of 0.1 mL of 3 µg/mL sodium fluorescein (VWR Life Science) in PBS, a video consisting of 15 frames shot at a rate 3 frames/s was recorded though the instrument’s green-fluorescence optics.

Videos were processed and microfilariae detected as described below.

### Video processing

To amplify the subtle movements captured by this system for the purposes of human review, the video was processed with FFmpg software (FFmpg.org) to highlight only pixels that differ significantly between adjacent video frames.

**Fig. 2:**
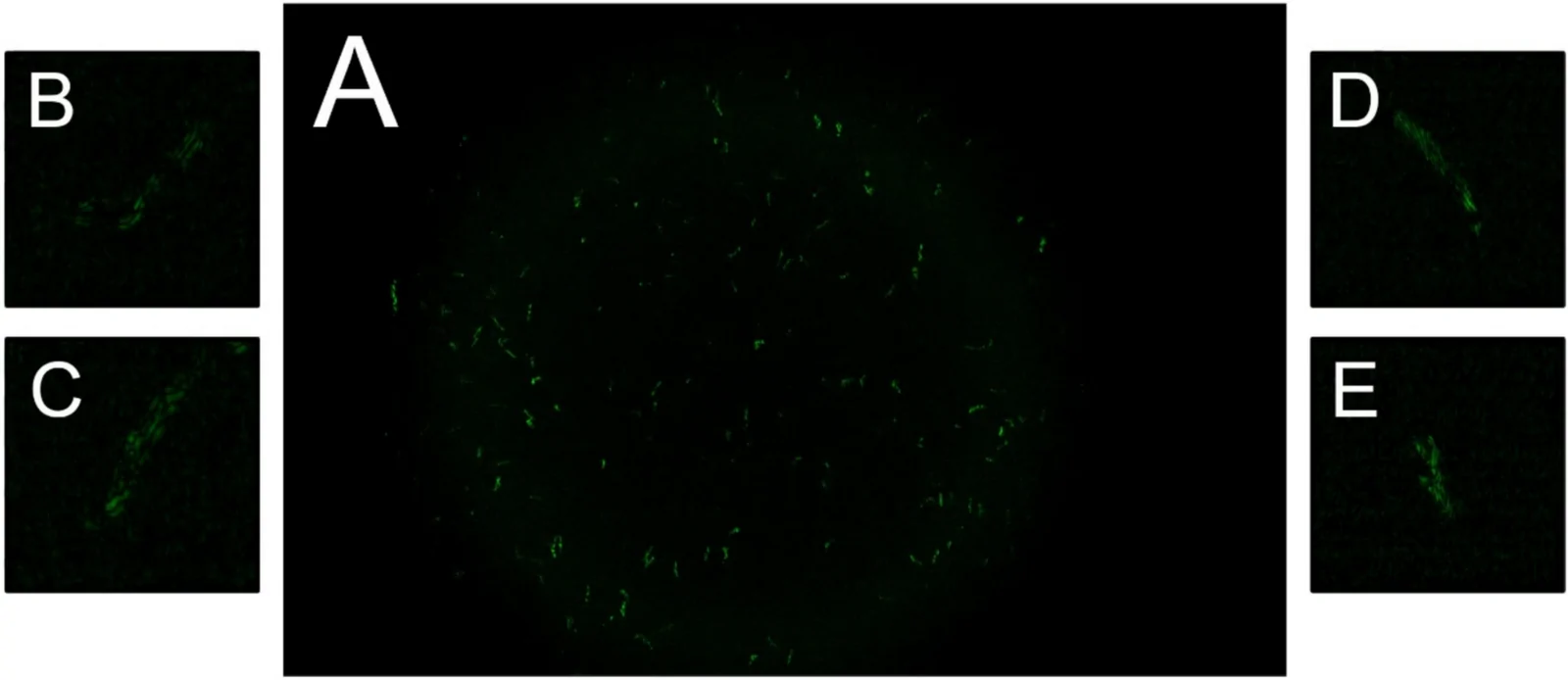
Still frames from a video from the All-in-One device after digital processing to enhance filarial movement. The images depict the surface of an entire sample chamber (**A**) as well as a number of individual motile microfilariae (**B**–**E**). The video used to generate this figure is shown in supplementary Fig. 1

Examples of single frames from difference-processed video are depicted in Fig. 2, which shows an image of both the entire SC mesh surface (Fig. 2A) as well as close-ups of individual microfilariae (Fig. 2B–E). The difference-processed video used to generate this still image is shown in Supplementary Fig. 1. In this case, raw video of the same individual microfilariae shown in Fig. 2B–E is shown to illustrate their appearance without video processing. Note that this enhanced video was not used for the automated detection of microfilariae but was instead for human review.

For detection, video was processed with OpenCV (www.opencv.org) an open-source computer vision library. Initially the video was processed using an implementation of the nearest-neighbors based background/foreground segmentation algorithm. This identifies moving objects within each frame by monitoring differences in the moving average values of each pixel’s channels.

Detected foreground (i.e., moving) objects in each frame were masked and the frame’s threshold set such that foreground pixels were white and background pixels were black. Then each frame’s average intensity (white value) was evaluated, and if it exceeded a set threshold it was discarded—such frames are indicative of broad-area difference caused by sudden light level changes or camera movement. The remaining frames were summed together to form a composite image of all foreground pixels in the video. Patches of white pixels that passed a size parameter filter were identified as microfilariae.

### Longevity

A sample containing approximately 40,000 mf/mL *D. immitis* was diluted with uninfected blood to produce 80 mL of a 1:80 dilution. Ten 1 mL aliquots were then analyzed and quantified with the AIO automated system and ten 20 µL drops were quantified manually as wet mounts (day 0).

The remainder of the stock sample was then stored at 4 °C and reanalyzed as above after 24, 28, 72, 96, and 144 h (i.e., days 1, 2, 3, 4, and 6, respectively).

### Quantification

A sample of infected blood containing *D. immitis* (~ 30,000 mf/mL) was diluted with uninfected blood at a ratio of 1:5. This was then used in a serial dilution with more uninfected blood to produce at least 7 mL each of 1:10, 1:20, 1:40, 1:60, 1:80, and 1:100 dilutions. Five 20 µL drops from each dilution were counted manually as wet mounts and five 1 mL aliquots were processed, analyzed, and quantified by the AIO automated system. Samples were stored at 4 °C during the entire process.

### Sensitivity and specificity

A sample of *D. immitis* infected blood (approximately 30,000 mf/mL) was diluted with uninfected blood at a ratio of 1:500. Some of this sample was further diluted twofold with uninfected blood to produce a 1:1000 dilution. Ten 1 mL aliquots of each dilution were then processed and analyzed with the AIO system as were ten 20-µL drops as manual wet mounts. In addition, ten aliquots of uninfected blood were also analyzed by both methods.

### Statistical analysis

Microfilaria counts from freshly received infected blood and from the same sample stored at 4 °C for various periods of time were compared against each other with a one-way analysis of variance (ANOVA) test using JASC statistical software (University of Amsterdam). Curves were fitted using Microsoft Excel.

## Results

**Fig. 3:**
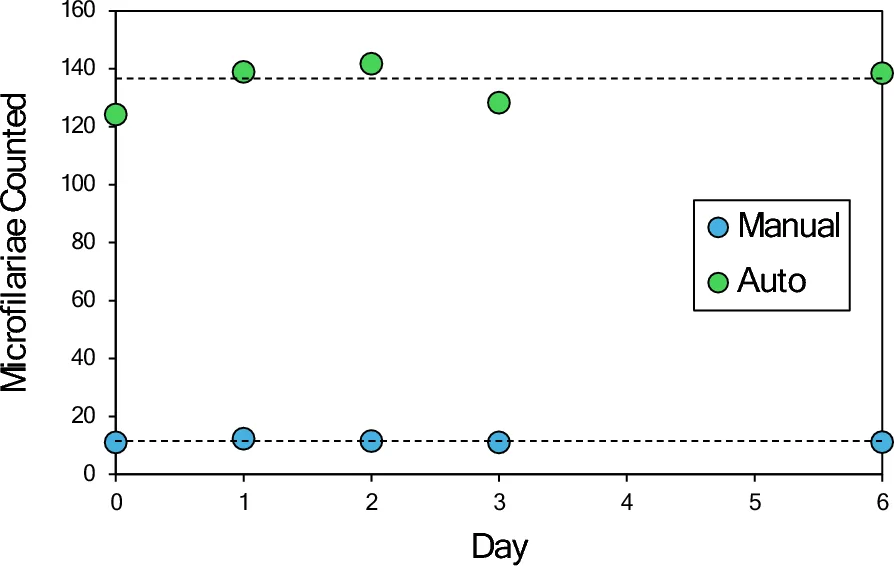
Lack of effect of blood storage on test counts. Microfilariae in ten subsamples of diluted, freshly received blood (Day 0) were counted by both the manual and automated methods and also subsequently on days 1, 2, 3, and 6. The average counts at each time point are shown. The two broken lines show the mean count for each method across the entire time course (manual = 11.4 microfilariae, automated = 138.4 microfilariae)

The large number of microfilaria counts required for this study could not feasibly be conducted in a single day. To allay concerns that counts may naturally decrease over the course of any given multi-day experiment owing to microfilarial mortality or morbidity, we conducted successive counts on the same sample stored at 4 °C over a period of 6 days using both manual and automated methods. There was no apparent change in the number of microfilariae detected by either method (Fig. 3), although qualitatively at day 6 the microfilariae appeared more lethargic with respect to the amplitude and frequency of their movements. After confirming equality of variances in the count results using Levene’s test (F_4,45_ = 1.165, *P* = 0.339), a one-way ANOVA analysis determined that there were no significant differences between the number of microfilariae counted on days 0 through 6 for both the manual (F_4,45_ = 1.388, *P* = 0.253) and automated (F_4,45_ = 0.74, *P* = 0.74) methods.

**Fig. 4:**
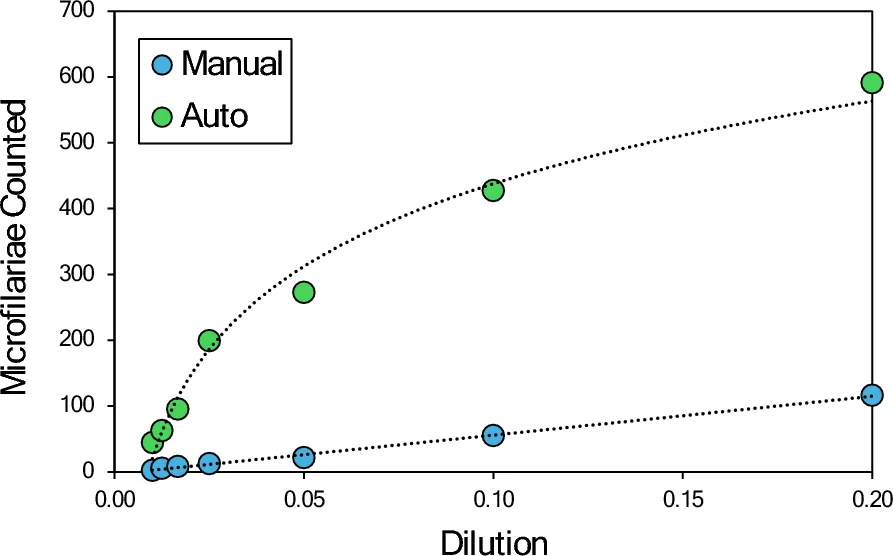
Titration of microfilarial counts. An infected blood sample was diluted with uninfected blood at ratios of 1:5, 1:10, 1:20, 1:40, 1:60, 1:80, and 1:100. Five subsamples of each dilution were counted by both methods and the means of each set plotted against the dilution. The manual counts were fitted by linear regression (*R*^2^ = 0.997) and the automated to a logarithmic curve (*R*^2^ = 0.9861)

To assess the relative magnitudes of counts between the automated AIO method and a wet mount, we produced seven dilutions of an infected sample with uninfected blood ranging from 1:5 to 1:100. Each of these samples were counted five times using both methods and the mean counts obtained plotted against their respective dilutions (Fig. 4). While the manual method exhibited a strong positive linear correlation with dilution magnitude (*R*^2^ = 0.9974), the automated did not; instead it exhibited a strong logarithmic relationship (*R*^2^ = 0.9861) that began to plateau at dilutions between 1:40 and 1:20. The automated method detected more microfilariae than the corresponding manual test at all concentrations.

**Fig. 5:**
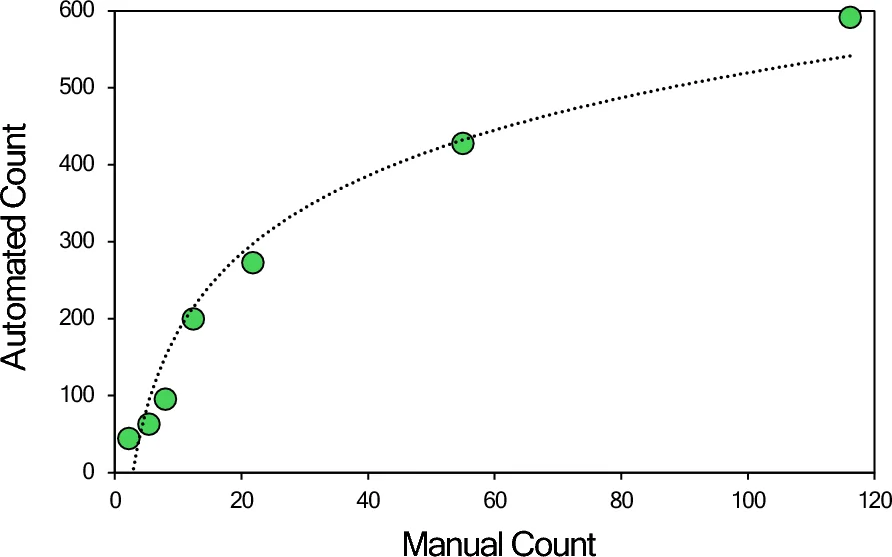
Correlation of manual and automated counts. The same data Fig. 4 were plotted against each other and then fitted to a logarithmic curve (*R*^2^ = 0.944)

**Fig. 6:**
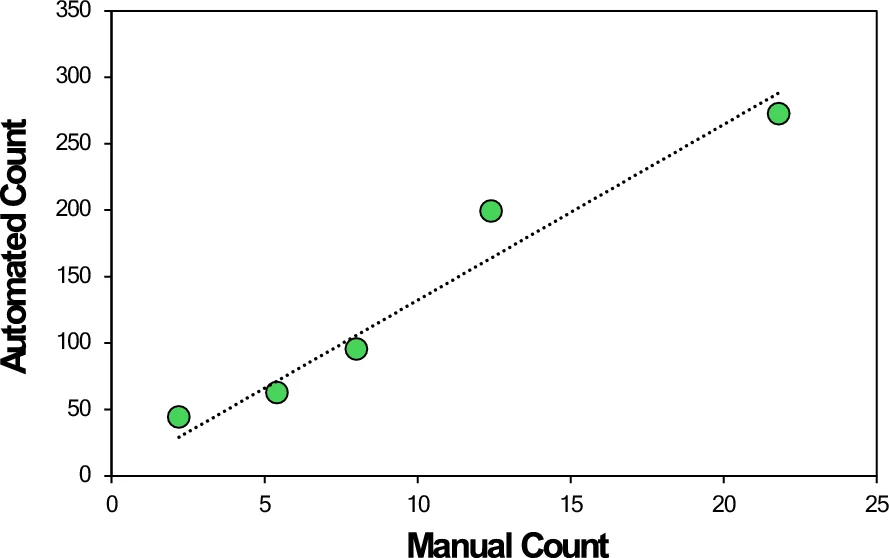
Linear range of count correlation. Manual and automated counts for samples diluted between 1:20 and 1:100 inclusive were plotted against each other and fitted by linear regression (*R*^2^ = 0.9851). The slope of the line is 13.2

Plotting the automated count results versus the manual produced a similar logarithmic curve (Fig. 5; *R*^2^ = 0.944); however, plotting only the five highest dilutions (1:20–1:100) produced a strong linear correlation (Fig. 6; *R*^2^ = 0.9851). The slope of the line (an indicator of the relative magnitudes of the counts from the two methods) was 13.2.

To assess the relative performance of the manual and automated methods at lower microfilariae levels two higher dilution samples (1:500 and 1:1000) were produced and each counted 10 times using each method. In addition, to assess specificity, a negative sample was also counted 10 times using each method.

Manual wet mounts detected at least 1 microfilaria in 6 of the 10 subsamples from the 1:500 dilution and in 1 of the 10 subsamples of the 1:1000 dilutions (Table 1). By contrast, the automated method detected microfilariae in all 10 of the subsamples from each dilution. Neither method detected microfilariae in any of the 10 negative blood subsamples.

**Table 1:** Sensitivity of the manual and automated methods

Dilution	Microfilariae counted										Mean	Sensitivity (%)
Manual method												
1:500	0	3	1	1	2	0	1	0	0	1	0.9	60
1:1000	0	0	1	0	0	0	0	0	0	0	0.1	10
Automated method												
1:500	5	5	11	10	15	3	10	7	8	8	8.2	100
1:1000	3	7	6	9	5	7	6	5	5	4	5.7	100

Ten subsamples of highly diluted infected blood (1:500 and 1:1000) were counted using both manual and automated methods. The numbers of microfilariae counted in each test are shown along with the mean count and resulting sensitivity

## Discussion

To maximize the likelihood of detecting infection, veterinary professional bodies such as the American Heartworm Society and the Companion Animal Parasite Council recommend that both antigen tests and microscopic methods are employed in diagnosis and that all dogs aged over 7 months are tested annually [9]. Unfortunately, microscopic examination of blood samples for microfilariae is inconvenient and time-consuming and the methods that are feasible in a clinical setting (such as smears and wet mounts) are not particularly sensitive [9]. These inconveniences and drawbacks present practical barriers to the widespread adoption of this dual-testing recommendation.

With this in mind, we sought to leverage the capabilities of the Parasight AIO parasitology analyzer coupled to the distinctive motility of *D. immitis* microfilariae to develop an automated microfilarial detection test that would enable simple and rapid in-house analysis and therefore encourage compliance with current testing guidelines.

Since the AIO’s optics are based on fluorescence imaging and not visible-light microscopy, we needed a suitable fluorescent light source to illuminate any microfilariae present on the surface of the SC mesh. To this effect we used a dilute solution of sodium fluorescein since the green light generated by this fluorophore could pass through the emission filter integrated into the instrument’s lens and so produce images on the camera sensor. After loading a sample onto the mesh and washing to remove the bulk of the erythrocytes we were able to observe moving microfilaria on the surface of the mesh (Fig. 2 and supplementary Fig. 1).

Although *D. immitis* microfilariae are only ~ 6 µm wide [32] and the magnification of the AIO optical column is only 1×, its high resolution lens and sensor provided sufficient resolving power (2.5 µm) to detect the microfilariae by virtue of the distortion of the underlying mesh as light was refracted through them.

Raw microfilariae videos were of low contrast and the moving microfilariae were difficult to detect both computationally and with the naked eye (supplementary Fig. 1). However, when adjacent video frames were processed to highlight pixels whose values had changed significantly and then composited into a new video, microfilarial movement became readily apparent (Fig. 2). Such videos were now amenable to image analysis to detect and enumerate the number of microfilariae on the mesh.

To determine whether this test could provide a viable alternative to manual examination, we decided to evaluate its performance relative to a standard laboratory wet-mount, which is likely the most commonly used test in-clinic owing to its simplicity and relative rapidity. Furthermore, although current best practice concentrates on just the detection of microfilariae and not their quantification, we did not limit our investigation to solely this binary approach. The relatively recent development of anthelmintic drug resistance in *D. immitis* [33, 34] is likely to lead to a change in guidance to include quantitative testing so that resistance can be evaluated by subsequent post-treatment reduction tests to monitor drug treatment efficacy [35]. Such testing for gastro-intestinal helminths has been the standard for decades in pasture animals [36], where resistance has long been a concerning issue [37–39], and may also become standard practice with companion animals since the discovery of resistance in canine *Ancylostoma* hookworms [40, 41]. As a result, we also evaluated the relative quantitative natures of these tests with an eye to an eventual change in guidelines in response to emerging therapeutic resistance.

This study required a large number of manual counts that could not feasibly be conducted in a single day. Our concern with regard to staging experiments over time was that the performance of either test might deteriorate over time as microfilariae aged in a refrigerator and became more lethargic. To alleviate this concern, we took a large volume of fresh, infected blood, conducted multiple replicate manual and automated counts, and then stored the remainder at 4 °C. The same stock was recounted on subsequent days to determine whether the performance of either test varied over time. We found that there was no statistically significant difference in the counts on any day relative to the first (Fig. 3), although by day 6 the microfilariae had qualitatively become substantially more lethargic. On the basis of these results, all tests within any given experiment were conducted within 3 days of receipt of blood samples.

Blood samples containing different concentrations of microfilaria were generated artificially by dilution with uninfected blood. Five replicates of each of these dilutions were then counted by both manual and automated methods. Since the automated method analyses a full 1 mL of blood compared with the 20 µL used for wet mount analyses, it was unsurprising that it detected substantially more microfilariae at each concentration (Fig. 4). The manual method produced a strong linear correlation across the dilution range; by contrast, the automated method exhibited a logarithmic relationship with dilution and lost linearity as the microfilarial concentration increased and the respective counts began to plateau. This occurred because increasingly more microfilariae began to overlap and abut at higher concentrations and thus became more difficult to discern individually. As clumps of microfilariae grew larger, entire clumps began to be ignored by the detection algorithm because of their size; consequently, the proportion of total microfilariae counted relative to lower concentrations began to fall.

Unsurprisingly, the same trends were observed when the two sets of counts were plotted against each other (Fig. 5). The two methods correlated linearly up to a concentration of approximately 1000 mf/mL as assessed using the counts obtained from the wet mounts (count × 50; Fig. 6). The slope of the least-squares regression through the data was 13.2, indicating that on average the automated method counted over 10 × more microfilariae than the manual. This was consistent with the data obtained on longevity where the average of all counts for the manual method was 11.4, while that for the automated was 134.3 (11.8 × more).

While the automated method detected approximately 10 × more microfilariae within the linear range than the manual, it used 50 × more blood, suggesting an 80% loss during processing. One consequence of this observation is that the AIO test generates lower counts than the wet mount after applying a traditional arithmetic multiplication factor to convert raw counts to values in mf/mL. For example, at a 1:20 dilution in the quantification arm of this study, the wet mount counted an average of 21.8 microfilariae while the AIO method counted 272.6. Since the wet mount used 20 µL of blood, this value could be converted to mf/mL by multiplying by a multiplication factor of 50 (i.e., 20 µL × 50 = 1 mL). Similarly, the AIO count would utilize a multiplication factor of 1 (since 1 mL of blood is used in the test). Thus, for the above example (at 1:20 dilution) the count for the wet mount would be 1090 mf/ml (21.8 × 50), while that for the AIO would be 272.6 (i.e., one quarter that of the wet mount). However, this difference can be corrected for using empirical data such as those presented here to adjust the multiplication factor accordingly. This approach has been used in the gastrointestinal parasitology to convert counts generated by novel methods to units that are more familiar to veterinarians and parasitologists [30, 31].

In this case, the slope of the line depicted in Fig. 6 (i.e., 13.2) can be used to calculate an multiplication factor of 3.8 (i.e., 50/13.2) that would convert the AIO raw count into a “wet mount equivalent” of 1036 mf/mL. Since counts from different analytical methods do not always scale simply with the volume of blood used [9], it should be noted that different empirically derived multiplication factors would be needed to report counts in, for example, “Knott test equivalents” or “thick smear equivalents.” Furthermore, any future changes or improvements to the AIO and/or its underlying algorithm would require its recalibration with respect to multiplication factor.

Since automated gastrointestinal nematode ova counts generated by the AIO device also generate higher counts than most manual methods and are correspondngly more sensitive than them [30, 31], the larger numbers of microfilariae detected by the AIO versus the thick smears suggested that the automated method would also be more sensitive than the manual. We confirmed this by conducting replicate counts on even higher dilutions of infected blood. At a 1:500 dilution (approximately 50 mf/mL as assessed from the average wet mount counts), the wet mount detected microfilariae in 6 of the 10 subsamples processed, while the automated method was positive in all 10 cases. At a 1:1000 dilution, only one of the 10 manual counts gave a positive result while all ten of the automated counts were still positive (sensitivity 100%). Both methods failed to detect microfilariae in 10 counts of a negative sample (specificity 100%). Since we did not have access to other filarioid species, and since we do not think it likely that this test will be able to distinguish between them, it should be noted that in this context “specificity” refers to the detection of filarioids in general and not to *D. immitis* specifically.

These data suggest that the automated Parasight AIO test is a suitable alternative for in-clinic manual wet-mounts (and likely other manual methods that also use very low volumes of blood). While we did not conduct a formal study with respect to test times, our experience was that careful counting of a wet mount slide at 100× (including the liquid that has seeped beyond the edges of the coverslip) took between 5 and 10 min. This was comparable to the 6 min it took from pouring the sample to obtaining a result with the automated method; however, since the automated method is essentially hands-off after sample pouring, it liberates analysts to conduct other tasks in the interim, which is not the case for manual methods. This, coupled with its substantially enhanced sensitivity, should free clinicians to follow current best practice without the associated inconvenience.

A disadvantage of the high sensitivity of the automated method is that it has a lower dynamic range than the wet mount (Figs. 4 and 5), and presumably to other manual methods. This might become problematic if/when reduction counts become standard practice, since pre-treatment counts from animals with high microfilarial burdens (> 1000 mf/mL) would not be sufficiently accurate to give a baseline for subsequent reduction tests and would generate artificially elevated metrics of drug resistance. We are currently developing algorithms that will detect cases where overlapping microfilariae are suppressing counts to alert clinicians to the issue so that the sample can be retested using a lower volume of blood to obtain a more accurate pre-treatment baseline. In addition, deep-learning-based classifiers present another approach toward filarial detection that may be more capable of distinguishing juxtaposed microfilariae. We are currently exploring the possibility of broadening the dynamic range of the current test using this approach.

Another disadvantage is that this method is unlikely to distinguish between *D. immitis* and *D. repens*—with the latter being more zoonotic. While this may not be as important in North America where *D. repens* is relatively rare, this is not the case in Europe [42]. However, distinguishing between these two species morphometrically through a conventional microscope is not entirely trivial, and may be beyond the capabilities of some or many analysts at veterinary practices. However, following current guidelines by also conducting antigen testing should inform clinicians about the true nature of any given infection. Lack of discrimination is also the case with the non-pathogenic cutaneous filarial worm *Acanthocheilonema reconditum*. While its microfilariae are slightly narrower than those of *D. immitis* (approximately 4.5 µm versus 6.3 µm) [32], it seems unlikely that this difference will be sufficient to reliably distinguish between them in this automated system. However, *A. reconditum* microfilariae are more motile than those of *D. immitis* in that they can readily be observed translocating across the substrate under the microscope. The possibility exists, therefore, that *A. reconditum* might be distinguished from *D. immitis* and *D. repens* on the basis of this behavioral property. This is a matter worthy of further investigation.

One shortcoming of this study is that it used blood from a limited number of donor animals and from experimentally infected dogs. While we do not think that any physicochemical/physiological differences between donors are likely to significantly affect the filtration properties of blood in this system, and thus the results, testing samples from a broader array of donors as well as from naturally infected animals will allow us to confirm this suspicion.

As discussed above, the data presented here suggest that the AIO method detects approximately 20% of the microfilariae added to the mesh of the SC. It is unclear at present how much of this is owing to actual losses during processing such as loss of some microfilariae though the capture mesh of the SC. Despite this, a large part of the discrepancy is unquestioningly due to limitations in the system’s optics (with respect to depth-of-field) and variations in the flatness of the SC mesh. For these reasons, the current system cannot capture the entire egg chamber at full focus at a single focal length. When taking still images of ova and oocysts, the system takes a number of images at different focal lengths to capture the entire surface area. As a result, the videos generated in this study only capture half of the surface area in full focus, making the detection of microfilariae in the other half either problematic or impossible. We are currently working on shooting two or more videos per test to fully capture the mesh surface, which should at least double the recovery and further enhance the sensitivity of the test.

Once the system is fully optimized in this respect, we plan to compare its performance against the arguable “optimal standard,” the Knott test, as recommended by the American Heartworm Society and the Companion Animal Parasite Council.

## Conclusions

We have developed a new, rapid, automated test to enumerate the number of *D. immits* microfilariae in the blood of heartworm-infected animals. The test requires minimal hands-on time and is over 10-times more sensitive than a standard 20-µL wet mount. Moreover, in-clinic immunoassays cost practices approximately US$ 20–30 each in the USA, while in-clinical microscopic methods cost approximately US$ 10–15 (assuming a technician salary of US$ 30 and a test-time of 20–30 min). By contrast, the AIO test retails at under US$ 10, making it a commercially viable alternative to manual examination of blood samples and a cost-effective adjunct to molecular testing. These economic considerations, coupled to the AIO’s ease-of-use and enhanced performance, could incentivize more clinicians to embrace current best-practice guidelines when testing for heartworm infection by using both immunological/molecular and hematological methods.

## Supplementary Information

Supplementary material 1.

## Data Availability

All data generated or analyzed during this study are included in this published article.

## Abbreviations

PBS: Phosphate buffered saline
AIO: All-in-One
mf/mL: Microfilariae/mL
SC: Sample chamber
